# Artificial Intelligence in Health Professions Education: Qualitative Study of Student Experiences

**DOI:** 10.2196/82432

**Published:** 2026-04-02

**Authors:** Marina Guirguis, Salomon Fotsing, Joanne Fevry, Christine Landry, Diane Bouchard-Lamothe, Jennifer Lacroix, Alireza Jalali

**Affiliations:** 1 Faculty of Medicine, University of Ottawa, Ottawa, ON, Canada; 2 Francophone Affairs, Faculty of Medicine, University of Ottawa, Ottawa, ON, Canada; 3 Department of Family Medicine, University of Ottawa, Ottawa, ON, Canada; 4 Institut du Savoir Montfort, Ottawa, ON, Canada; 5 School of Pharmaceutical Sciences, Faculty of Medicine, University of Ottawa, Ottawa, ON, Canada; 6 Department of Innovation in Medical Education, Faculty of Medicine, University of Ottawa, 451 Smyth (3247BD), Ottawa, ON, K1H 8M5, Canada, 1 613-562-5800 ext 8117

**Keywords:** artificial intelligence, AI, qualitative study, health professions education, learning technologies, ChatGPT, learning experience, digital literacy

## Abstract

**Background:**

Artificial intelligence (AI) is increasingly integrated into education and health care, raising questions about how students use these technologies and how AI influences their learning. In health education, understanding these trends is particularly important because student learning directly impacts future clinical skills.

**Objective:**

This study aimed to explore the use of AI tools by health sciences students at the University of Ottawa. More specifically, it sought to identify the most frequently used AI tools, describe students’ usage habits, determine which tools support knowledge acquisition and skill development, and gather students’ recommendations for effective strategies to raise awareness and train their peers on the responsible use of AI.

**Methods:**

A qualitative approach was used with students from 10 health professions who reported using AI in their studies. Data were collected through semistructured interviews and an open-ended qualitative online survey. Inductive thematic analysis within an interpretive paradigm was applied to capture patterns, perceptions, and emergent themes.

**Results:**

A total of 51 health professions students participated in the study. Most were women between the ages of 20 and 29 years. ChatGPT (OpenAI) emerged as the most frequently used AI tool. Students perceived AI as a complementary tool that facilitated knowledge acquisition, skill development, writing, and problem-solving. AI adoption was driven by curiosity, peer influence, and the desire to improve work efficiency. Students critically evaluated AI results, integrated the tools into their learning processes, and emphasized the importance of technical skills, critical thinking, and digital literacy. Peer learning, hands-on demonstrations, and access to online resources were recommended for effective AI training.

**Conclusions:**

This research demonstrates that health professions students actively use AI tools, particularly ChatGPT, to support learning, skill development, and academic tasks. Although AI is valuable as an educational aid and its use varies by student and context, this highlights the need for structured guidance, critical evaluation skills, and peer-supported training. These findings highlight the importance of thoughtfully integrating AI into educational programs to enhance learning outcomes, foster skill acquisition, and ensure responsible and effective adoption.

## Introduction

Presently, the use of artificial intelligence (AI) has become commonplace in health care, health professions training, and the pharmaceutical industry. AI refers to the capacity of machines to simulate human functions such as reasoning, learning, and problem-solving through algorithms [1,2]. This technology, which “mimics human cognitive functions,” is becoming increasingly effective in the health care sector. Although it sometimes raises ethical concerns [3], AI now assists clinicians in making better decisions, especially in fields like diagnostic imaging, where it aids in image interpretation [2,4], and dermatology, where it helps in the early detection of skin cancer by using efficient classifiers for skin tumors [5]. In addition, in the pharmaceutical field, AI accelerates drug design and discovery processes [2,6].

The use of AI in health professional education is also growing. With the aid of tools like ChatGPT (OpenAI), it can create simulated clinical environments, evaluate clinician-simulated patient communication, create quizzes, and develop curricula [7]. At the same time, it can respond instantly to specific questions related to students’ areas of study through AI chatbots used in medical education [8]. The new generation of health learners is much more inclined to adopt and use this technology. In 2022, a national Canadian survey of nearly 2000 health professions students showed that AI was viewed favorably by students, who also believe that this technology will positively influence their future careers [9]. Similarly, in March 2023, a systematic review reported that health professions students have a favorable attitude toward the use of AI [10].

From an academic standpoint, the use of AI by health professions students increasingly raises the question of the “learning experience.” Learning is a dynamic concept defined as a process that allows individuals to perceive objects, interact with them, and integrate them into their social, cognitive, and emotional dimensions to transform, create, or evolve their cognitive structure [11]. The learning experience goes beyond knowledge acquisition. It facilitates the development of skills and cultural values through observation, imitation, trial, repetition, and presentation [12]. Therefore, an important question regarding AI in health professions training programs is how AI contributes to the students’ learning experience. Shorey et al [13] scoping review of the learning styles, preferences, and needs of Generation Z health students marks a first step in this direction. Given the increasing adoption of AI, raising awareness and providing training on the proper use of these tools has become essential [9]. Peer education is a viable approach because it allows students to share valuable insights and fosters a collaborative learning environment that encourages peers to use AI tools [14,15].

Access to AI tools is also an important element in the training of health science students, given their increasing use in modern learning and clinical reasoning processes [16]. However, socioeconomic and linguistic factors may influence their use by these students, as some of these tools are mainly programmed in English and sometimes require payment to access more advanced features [16,17]. In a bilingual institution of higher learning such as the University of Ottawa, where courses are taught in French, English, or both, it is essential to ensure equitable access to these resources for all students.

This qualitative study aimed to explore which AI tools health professions students at the University of Ottawa report using, describe their usage habits, identify the tools that help them acquire new knowledge and develop skills, and explore what these students consider to be the best strategies for raising awareness and training their peers on the proper use of AI in learning environments. Specifically, we focused on how students use AI tools to support their learning and on strategies they recommend for peer awareness and training.

## Methods

### Qualitative Approach and Research Paradigm

An inductive thematic analysis approach, following an interpretive research paradigm, was used to identify the AI platforms most used by health professions students during their learning activities. This approach was chosen for its relevance to explore perceptions and experiences regarding AI tools in medical education [18]. This approach, based on the interpretive paradigm, posits that participants’ narratives construct meanings, which promotes a detailed understanding of behaviors and points of view [19]. Therefore, in accordance with the research objectives, the inductive approach facilitated the emergence of themes directly from the data for a more flexible and data-driven interpretation [20]. This paper was written by using the Standards for Reporting Qualitative Research (SRQR) guide (Checklist 1) [21].

### Research Characteristics and Reflexivity

The research team was multidisciplinary and followed a collaborative approach, demonstrating reflexivity at each phase of the study. Team members included MG, an undergraduate medical student experienced in qualitative research, who contributed to study design, data collection, thematic analysis, and writing; SF, a physician and clinical research manager, who provided methodological guidance, qualitative analysis, results interpretation, and manuscript revision; JF, an anatomy educator with expertise in qualitative research and scientific writing, supported data coding, thematic analysis, and writing; CL, a clinician-researcher and pharmacy program director, offered insights into results interpretation; DB-L, a medical education advisor, contributed to the interpretation of the results and the revision of the manuscript; JL, a program manager experienced in qualitative analysis, helped organize and coordinate the project; and AJ, a physician and anatomy professor with expertise in technology-assisted learning and educational innovation, contributed to data analysis, results interpretation, and manuscript revision.

Regular meetings encouraged open dialogue, allowing all coauthors to critically reflect on their assumptions, disciplinary lenses, and interpretations throughout data collection, analysis, and manuscript writing.

### Context

Our study was conducted among health professions students at the University of Ottawa. All data were taken outside regular class hours, either through online interviews on Zoom or via an asynchronous online questionnaire.

### Sampling Strategy

A nonprobabilistic sampling method was used, following the recommendations of Murphy et al [22] and Higginbottom [23]. Only students who used or had previously used AI platforms for their learning activities in the previous academic year were eligible. To identify eligible participants, all students in the targeted programs received an invitation email including a brief screening question asking whether they had used AI tools (eg, ChatGPT, Perplexity AI, or any other AI-assisted learning platforms) for their academic activities in the last academic year. Only those who answered “yes” were invited to participate in the study, while students who responded “no” were excluded.

The targeted programs included medicine, pharmacy, molecular and translational medicine, nursing, occupational therapy, physiotherapy, audiology, speech-language pathology, health sciences, and psychology. Our target sample size was based on the writings of Guest et al [24], which specified that in a qualitative study, fundamental elements of the themes studied are present in participants’ discourse after 6 interviews, and theoretical saturation of information is achieved after the first 12 interviews. Therefore, we planned to recruit a minimum of 6 participants from each training program, with no restrictions on the maximum number of participants. The distribution of students by program was not tracked separately for the interviews and the questionnaire; overall characteristics of participants are presented in Table 1.

**Table 1.** Sociodemographic characteristics of participants (N=51).

Category	Values, n
Age (years)	
<20	9
20‐29	39
30‐39	2
40‐49	1
≥50	0
Gender	
Women	41
Men	9
I prefer not to answer	1
Others	0
Health professions	
Health sciences	27
Medicine	8
Psychology	8
Nursing	1
Pharmacy	1
Others	
Biomedical sciences	5
Biology	1

### Data Collection

Data collection began after the recruitment phase, between January and March 2024. Invitation emails to participate in the research project were sent to all students in the targeted programs. Those who agreed to participate were met for semistructured online interviews via Zoom, lasting 30‐45 minutes each. These interviews were conducted according to an interview guide developed in advance by the research team (see Section A of Multimedia Appendix 1). The guide aimed at capturing information on the most used AI platforms by health professions students at the University of Ottawa, their usage habits of these platforms, the platforms that they believed allowed them to acquire new knowledge and develop skills, and the best strategies for raising awareness and training their peers in the appropriate use of AI in learning.

The guide also included short vignettes to help organize questions about learning processes with AI tools. These vignettes, composed of a definition of the learning process [25,26] and Kolb’s [27] experiential learning model, are fully incorporated into the guide provided in Section A of Multimedia Appendix 1.

However, after only 6 interviews over a 4-month period, data collection was adapted to include an online questionnaire using the same interview guide. A total of 51 students made up the final sample after 6 students participated in semistructured interviews and 45 students completed the online qualitative questionnaire. The interview guide and the online questionnaire were made available in both French and English to accommodate participants’ language preferences and diffused between June and July 2024. The interview guide was reproduced identically on the online questionnaire platform (SurveyMonkey), thus ensuring that the same questions were asked in both data collection methods while ensuring methodological continuity. New invitation emails were sent to all students in the targeted programs.

### Data Processing and Analysis

#### Interview Process

The semistructured interviews were conducted on Zoom. No questions that could identify participants were asked. The audio of the interviews was recorded and transcribed using the WhisperAI transcription application. The transcriptions were then verified by a member of the research team for accuracy. To protect anonymity, the transcripts were saved in the order in which the interviews were conducted, and no identifiers were used in the file names. Moreover, interviews and transcriptions were conducted by two separate individuals. All files were password-protected.

#### Survey Process

Following ethics modification, a qualitative online questionnaire designed to elicit narrative and open-ended responses was hosted on the SurveyMonkey platform. Only one optional question allowing for the identification of participants was asked: “participants could provide their email address at the end of the questionnaire if they wished to be included in a draw for one of five gift cards as compensation.” To protect anonymity, participants’ responses were saved in the order in which they were received and were coded (eg, P1 for participant 1). The responses were stored in a password-protected Microsoft Excel file and kept separate from the email addresses, which were also in a password-protected Microsoft Excel file.

During the analysis, the information collected during the interviews and questionnaires was treated together as a single qualitative corpus. All coding was performed inductively; although Kolb’s experiential learning model informed the development of the questions, it did not guide the coding process. Each participant’s questionnaire responses were treated as individual transcripts and coded using the same inductive thematic analysis procedure as the interview data. Transcripts were read and revised several times to identify patterns and themes emerging from the data. To reinforce the methodological rigor of our qualitative study, a double thematic analysis was carried out. First, a researcher identified and categorized emerging themes from the corpus of data. Subsequently, a second researcher went back over all the material independently to reproduce the thematic analysis. This approach is part of a logic of analytical triangulation, aimed at limiting interpretive bias and reinforcing the credibility of the results as recommended by Paillé and Mucchielli [28]. The confrontation of analyses enabled us to discuss divergences, refine thematic categories, and arrive at a consensual interpretation of the material.

### Techniques to Enhance Trustworthiness

To ensure the trustworthiness of the analysis, the data were analyzed systematically. An experienced qualitative researcher manually coded 100% of the data, and a second member of the research team independently coded 25% of the dataset. This second coder was trained by the first to master the principles of qualitative analysis and the steps involved in coding interviews and to ensure accurate coding. The second coder coded two randomly selected complete transcripts and half of a third. The two coders then reviewed and compared their coding, and clarifications were made to better represent the participants’ experience until consensus was reached. No formal Cohen κ or percent agreement was calculated; reliability was ensured through this consensus-based process.

Next, a tree diagram was created to organize the coded data into potential themes based on observed similarities. Latent thematic analysis, using an interpretive approach, was used to identify subthemes. Finally, the two coders discussed the themes and subthemes related to the selected quotes. The coding framework was developed using NVivo software (version 12; QSR International) [29].

### Ethical Considerations

This study was reviewed and approved in accordance with the standards of the Research Ethics Board (REB) of the University of Ottawa (#H-11-23-9455). All participants provided informed consent prior to data collection, which took place outside regular class hours. Due to a low recruitment rate, a modification of the ethics protocol was requested and approved by the REB to allow participants to complete an online questionnaire instead of taking part in interviews. This modification was implemented during the data collection phase. Compensation was offered in the form of an optional draw for one of five CAD $25 (US $18.08) gift cards.

Participant privacy and confidentiality were strictly maintained throughout the study. All data were anonymized using participant codes, and no identifying information was included in the transcript or analysis. All data were securely stored and accessible only to the research team.

## Results

### Participant Characteristics and Qualitative Findings

In total, 51 students from various health-related fields who had used AI tools during their training activities participated in this study. Most participants were between 20 and 29 years old (n=39). In terms of gender identity, the majority identified as women (n=41), followed by men (n=9), and 1 participant preferred not to disclose. No participants identified as nonbinary or transgender persons.

Health sciences was the most reported field of study (n=27), followed by medicine and psychology, each with 8 participants (see Table 1).

Participants reported using a wide variety of AI tools. ChatGPT was by far the most frequently used, with 49 respondents indicating its use. Other tools mentioned included Microsoft Copilot (n=2), Perplexity AI (n=2), Anthropic Claude (n=2), and several specialized AI tools, such as Google Bard AI, Summarizer AI, Grammarly AI, Midjourney, and Scite, each cited by one respondent (see Section B of Multimedia Appendix 1 for further details).

**Figure 1.**
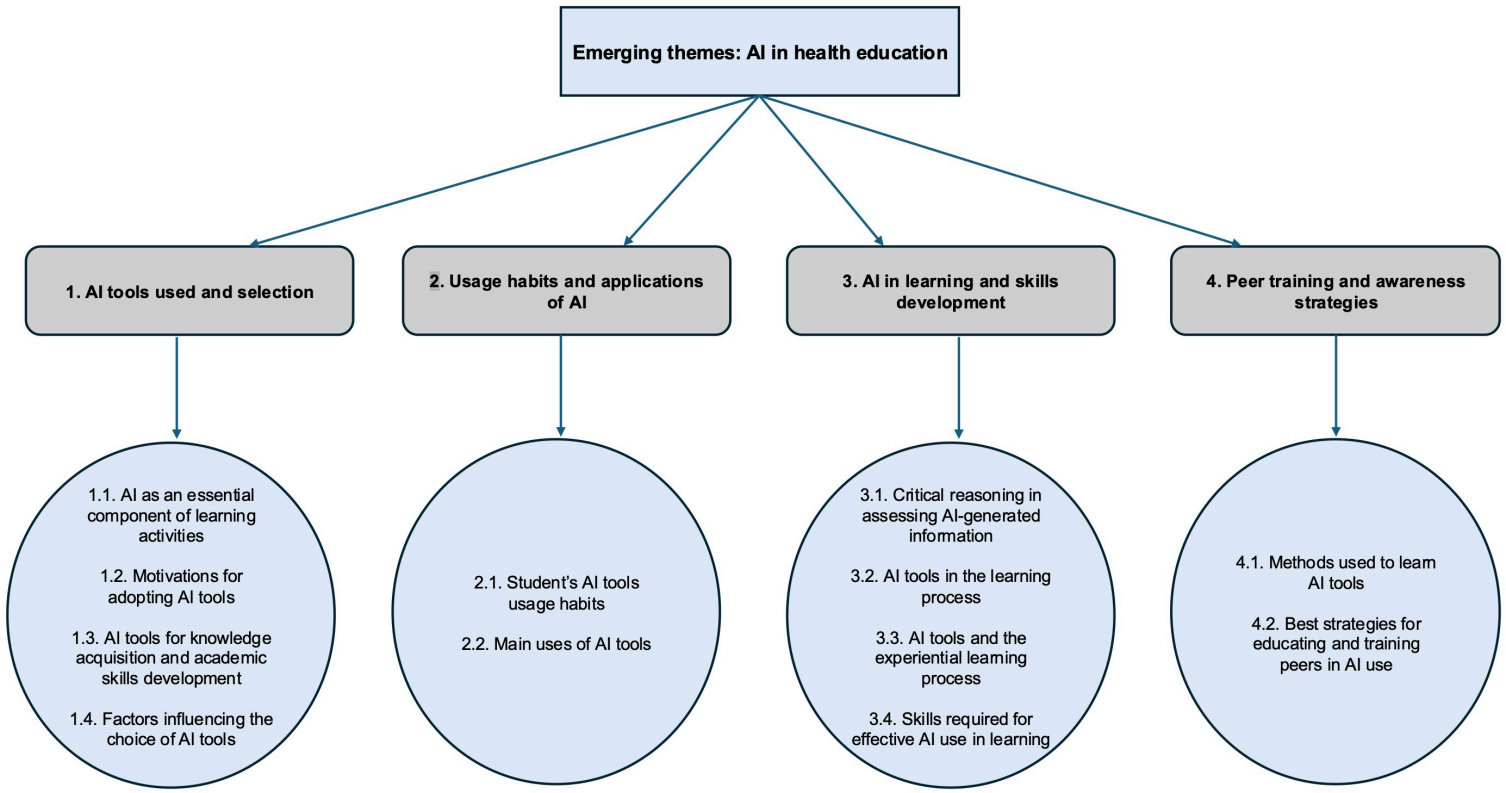
Concept map illustrating the emerging themes and subthemes of the qualitative data analysis. AI: artificial intelligence.

Themes were identified and developed from the content of the semistructured interviews and the responses to the qualitative survey, then organized into 4 main thematic areas (see Figure 1 for a summary).

### Theme 1: AI Tools Used and Selection

#### Theme 1.1: AI as an Essential Component of Learning Activities

Health care students have varying opinions about the role of AI in their learning activities. Among 51 respondents, 30 rated AI as essential or very beneficial. These students highlight its ability to enhance learning by helping to organize course materials, provide quick answers, and personalize educational experiences. For example, one participant illustrated this by stating:

*I have used ChatGPT to help summarize papers and great sample questions and to read my work and compare it to the rubric.*

[Participant 38]

They highlighted its usefulness in saving time and improving learning through tailored explanations and help with challenges like clarifying complex ideas or improving writing. As one participant noted:

*Yes. I think it is an essential component because it can offer tools to approach/view your research in new ways. Therefore, it can aid in overcoming any hurdles, like feeling stuck when writing.*

[Participant 23]

However, 21 respondents did not perceive AI as essential. Many of them regarded AI as a complementary aid rather than a primary resource, recognizing its usefulness while questioning its necessity. As one respondent noted:

*I don't consider it to be essential, but I consider it to be helpful.*

[Participant 19]

Concerns were raised about the reliability of AI, with one participant citing the presence of incomplete or inaccurate information on online platforms. Others reported using AI for targeted tasks but did not consider it integral to their overall learning process. A few respondents had only recently begun using AI and, although they acknowledged its potential, did not yet view it as indispensable. For example, one participant explained:

*I only started using AI recently and I would not necessarily say it is essential component but rather an assistant to my learning...It is more of a helper than essential at this point in my journey.*

[Participant 25]

#### Theme 1.2: Motivations for Adopting AI Tools

Curiosity was a key factor. Some respondents were intrigued by the widespread debate and excitement surrounding AI, particularly ChatGPT. Often encouraged by others, they were motivated to explore AI by the desire to see how it could be useful in their academic and professional lives.

A few respondents (n=2) were early adopters and experimenting with AI from its initial stages. One began exploring ChatGPT at its initial public launch, driven by curiosity and social media exposure:

*Honestly, when I started experimenting with AI tools, especially ChatGPT, it was the first one I used, I think it was really in December 2022, during its first days of public access...it was out of fear, out of curiosity and I had heard about it...*

[Participant 1]

The influence of peers, colleagues, and professors was also significant for 15 respondents, who were encouraged to try AI after observing others using it or receiving recommendations. This indicates the role of social and professional networks in the adoption of new technologies. As one student noted:

*My friend suggested that I try it out for help.*

[Participant 48]

Another shared:

*My supervisor actually recommended it for my data analysis; they told me to use it when I am unsure of how to use Excel or SPSS to work with data.*

[Participant 35]

Practical considerations, such as improving efficiency, saving time, and enhancing convenience, motivated 14 respondents to adopt AI tools. They found AI helpful in streamlining tasks and making their work processes more manageable. One participant noted:

*The fact that it is an accessible tool and allows me to save time.*

[Participant 14]

Twelve respondents were also motivated to use AI for professional or academic needs, frequently to gain a better understanding of procedures or expectations before starting a task. Some students used it to make sense of academic processes, particularly while navigating an unfamiliar system. One respondent stated:

*I am an international student, and I was not familiar with many academic based process and AI could make them clear for me.*

[Participant 29]

Some respondents (n=5) turned to AI out of frustration with traditional search engines like Google, which they found unhelpful or overwhelming. They saw AI as a more responsive and accurate alternative. For example, one participant noted:

*The answers/explanations that Google offered me were vague and took longer to find.*

[Participant 7]

And another mentioned:

*When Google wasn't giving me the answers I needed or was overwhelming me and confusing me instead of helping.*

[Participant 43]

A few respondents were motivated by innovation, recognizing AI’s cutting-edge potential to make their work easier and more unique. One student explained:

*Innovation, being different and unique. Having my work done easier for me. Being ahead of the curve and being more innovative and productive*.

[Participant 49]

#### Theme 1.3: AI Tools for Knowledge Acquisition and Academic Skills Development

ChatGPT is the predominant AI tool used by health professions students for learning, mostly for the development of academic skills, such as refining data analysis, understanding complex topics, and enhancing writing abilities. For example, one participant mentioned:

*ChatGPT is good at helping me refine my data analysis. It helps me distinguish between certain nuances of data analysis etc. when I ask it questions.*

[Participant 25]

And another highlighted the importance of writing:

*I think it helps me to develop new skills in the writing process, including synthesizing information or teaching me how to write in a more concise manner.*

[Participant 28]

However, some respondents reported not using AI tools for learning, with a few indicating unfamiliarity with these technologies, falling into the “none or N/A*”* category.

Beyond ChatGPT, other AI tools were also mentioned for their specific functionalities. Wolfram was praised for its mathematics capabilities, text-to-speech models for aiding study through text-to-speech, and Consensus for summarizing research papers. One student stated, highlighting the tool’s role in academic research:

*...I also like how Research Rabbit (I think it’s AI?) helps find links from one paper to others that are similar, it makes literature searching much easier.*

[Participant 23]

Finally, language learning proved important for some, as these tools helped improve grammar and clarity. As one student noted:

*When AI helps me rephrase my sentences to make them clearer, it allows me to acquire new knowledge or skills on the grammatical side.*

[Participant 12]

#### Theme 1.4: Factors Influencing the Choice of AI Tools

Most health professions students did not explore multiple AI tools before choosing their current ones. Only 6 out of 51 respondents experimented with different tools. However, 23 respondents shared insights into their choice, highlighting several key factors.

Ease of access was a significant factor, with 10 respondents preferring tools that are free or provided by educational institutions. These tools were chosen for their simplicity and minimal setup requirements:

*I have free open access via my institution.*

[Participant 24]

*It is simple to use. I would be open to trying other tools, but when the tools require me to set up a whole account, pay, or use personal data to use them, I find this to be a deterrent.*

[Participant 28]

Specific features also influenced the choice, with 7 respondents valuing functionalities such as summarizing scientific papers or handling documents:

*I chose Consensus for literature synthesizing because it presented the information in the best manner and is set up to aid in research specifically.*

[Participant 23]

One participant characterized the tool’s benefits as “bilingual” [Participant 16], in addition to being free, rapid, and efficient, while also emphasizing the necessity of using the tool judiciously.

Familiarity played a role for 6 respondents, who chose tools they were already aware of, highlighting a preference for sticking with known options. One participant mentioned:

*I started with ChatGPT as that is the one I kept hearing about. It’s suited my needs thus far as I don't use it very often. If I hear of something better, I might explore that too.*

[Participant 19]

Popularity influenced 5 respondents, who opted for widely recognized tools, believing them to be reliable. As one student noted:

*ChatGPT seemed the most popular.*

[Participant 37]

Finally, ease of use was crucial for 5 respondents, who favored tools that were user-friendly. Less commonly mentioned factors included perceived reliability, speed, and efficacy, underscoring the value of dependable and efficient tools. One student explained:

*I chose ChatGPT because it seems the most reliable and I have had no reason to seek other tools.*

[Participant 34]

### Theme 2: Usage Habits and Applications of AI

#### Theme 2.1: Students’ AI Tools Usage Habits

Health professions students showed varied practices regarding the frequency and contexts of AI tools use, focusing on educational support, writing assistance, and information retrieval. Many respondents (n=19) use AI to aid in understanding complex concepts, studying, and verifying knowledge, which indicates how these tools can enhance learning. As one student explained:

*I use them when I don’t understand homework questions or need to double check an answer.*

[Participant 33]

Writing assistance is another significant use, with 13 respondents relying on AI for drafting, revising, and brainstorming ideas. AI is used not only in academic writing but also in professional communication, such as writing emails. As one participant noted:

*For each of my courses that I undertake and also alongside my professional life, whether it’s for writing emails or other things.*

[Participant 2]

This highlights the versatility of AI in supporting various writing tasks. Some respondents, however, use AI infrequently or lack clear usage habits. Nearly 11 respondents expressed uncertainty about their AI usage, suggesting they are still exploring the tool’s potential or have not fully integrated it into their routines. One student shared:

*Very infrequently used so I do not have habits for use.*

[Participant 48]

AI is also used for general information retrieval and research, with 7 respondents using it for quick access to relevant information. As mentioned by one participant:

*I use it as a shortcut every time I do research or need information.*

[Participant 14]

A small number of respondents use AI for specific tasks like coding or data management, demonstrating AI’s value in specialized technical problem-solving. For instance, one student mentioned:

*When I am really stuck on how to do something in data analysis software, I ask ChatGPT for the best way.*

[Participant 35]

In terms of frequency, some respondents reported using AI daily, while others mentioned using it 1 to 3 times per week. The consistency of usage varied as well, with several describing their use as regular and others characterizing it as more occasional. This highlights the flexible nature of AI tools in meeting diverse needs.

#### Theme 2.2: Main Uses of AI Tools

Participants identified several common applications of AI tools, including in revision, writing, learning, research, and knowledge testing.

Revision and synthesis emerged as the most common use, with 28 respondents using AI to refine and organize information, edit text, and summarize complex material. AI is frequently used to structure ideas and enhance communication, including drafting emails and quizzing on notes. One student, for example, summarized their use as follows:

*...revising notes, synthetizing information.*

[Participant 41]

Writing is another significant area, with 22 respondents using AI for drafting, idea generation, and refining writing style. AI helps tailor content to specific tones and styles, enhancing both the creative and revision stages of writing. As one student mentioned:

*I mostly use these tools to proofread my writing or to enhance my writing style when I'm hit with “writer’s block.”*

[Participant 19]

For learning, 16 respondents rely on AI to clarify complex concepts, gain new knowledge, and validate their own reasoning. AI aids in understanding difficult topics and applying knowledge in practical scenarios, such as informing clinical practices in psychology. One psychology student noted:

*...I also use them to verify the clarity of my own writing. If I have an intervention in mind for a client (I am in psychology), I ask what research recommends for the name of whichever diagnosis I want to treatment plan for. I then double check that it aligns with what I read...*

[Participant 22]

In research, 8 respondents use AI to find specific answers, summarize articles, and navigate academic language. AI is valued for making research more accessible by bridging the gap between lay and academic terminology. One student explained:

*It is very useful to research topics when you don't know the exact language used in the literature, you can ask question in more common language and get to the information about the names of the theories and components that allows you to do real research to find the actual research papers relevant.*

[Participant 24]

Finally, knowledge testing involves using AI to create quizzes, check answers, and master complex tasks, such as data analysis in SPSS (IBM Corp):

*I use it to ask how to analyse data in a more complicated way in SPSS. My education on SPSS was simple and was able to guide me to do simple tasks, but my research is a bit more complicated.*

[Participant 35]

### Theme 3: AI in Learning and Skills Development

#### Theme 3.1: Critical Reasoning in Assessing AI-Generated Information

According to the survey analysis, students pursuing health professions use a range of critical thinking techniques to evaluate information produced by AI, indicating varying degrees of confidence and reliance on these technologies.

Cross-referencing AI outputs with credible sources is the most popular strategy, as reported by 29 respondents. They often compare AI-generated content with peer-reviewed articles, course materials, or trusted resources to verify accuracy, demonstrating a cautious approach to ensure that the AI information aligns with established knowledge. As one respondent noted:

*I compare it to peer-reviewed articles to see if the findings match up.*

[Participant 51]

Another prevalent strategy, mentioned by 16 respondents, involves using personal knowledge and common sense to evaluate AI content. These users rely on their prior understanding and logical reasoning to judge the validity of the information. For example, one respondent stated:

*I like to see whether it makes sense and matches up with notes taken in class and information I already have and know.*

[Participant 45]

Reviewing and editing AI-generated content is also common, with 17 respondents indicating that they scrutinize and revise AI outputs to ensure alignment with their expectations. This active engagement helps refine the content before it is used. As mentioned by one participant:

*I always read it over, take out things that don’t make sense or I don’t agree with, and then rewrite it myself.*

[Participant 46]

#### Theme 3.2: AI Tools in the Learning Process

AI tools play a nuanced role in the learning process, supporting dynamic engagement with knowledge through social, cognitive, and affective dimensions to develop cognitive structures.

##### Perception and Interaction With Information

Nearly 12 respondents noted that AI tools aid in recognizing and understanding information, enhancing the initial phase of learning. AI also facilitates interaction with data, with respondents emphasizing its role in manipulating and engaging with information crucial for deepening understanding. One student explained:

*I believe AI tools help to perceive objects from a different perspective, in order to better understand the material*.

[Participant 18]

##### Integration and Cognitive Development

A total of 12 respondents asserted that AI supports learners in embedding new skills and knowledge into their broader contexts, helping them integrate what they learn into their daily lives. Additionally, AI tools assist in transforming and refining cognitive structures, playing a key role in advancing cognitive development through learning processes. As one participant explained:

*I would say that AI tools allow to transform, to create, and to develop the cognitive structure. However, I would not say that AI tools are, in themselves, information or knowledge. They should rather be used to guide or confirm our information and our knowledge.*

[Participant 16]

##### Skills Development

AI’s impact on skill acquisition was acknowledged, though its influence on attitudes and values was considered minimal. Four respondents generally viewed AI as more effective in enhancing cognitive and skill-based learning rather than shaping affective outcomes. As one respondent noted:

*AI fits into the knowledge and information objects of learning. AI cannot teach new attitudes or values, but it can present new information and knowledge, as well as new ways to learn skills. I don't think it is advanced enough to help individuals perceive and interact with objects.*

[Participant 36]

##### Information and Knowledge Acquisition

The most frequently mentioned theme was AI’s role in acquiring and refining information and knowledge. A total of 23 respondents identified AI as a critical facilitator in these areas, underscoring its importance in the learning process. One student mentioned:

*I think AI fits in terms of knowledge acquisition and information gathering, both of which can help with learning.*

[Participant 48]

### Theme 3.3: AI Tools and the Experiential Learning Process

In our survey of health professions students on AI tools in education, we examined how these tools align with phases of experiential learning: concrete experience, reflective observation, abstract conceptualization, and active experimentation.

#### Concrete Experience

Overall, 13 respondents identified AI tools as fitting within this phase, where learners interact with new or known situations to gain direct experiences. Highlighting their role in engaging directly with problems, one comment mentioned:

*AI tools can be a great way to get experience and learn for example, if I am stuck in finding a code in R that gives me the statistical analysis I need. I can ask ChatGPT and troubleshoot with the code it gives me, study the code and integrate it into my bank of knowledge for next time.*

[Participant 27]

#### Reflective Observation

A total of 14 responses placed AI tools in this phase, where learners analyze and evaluate their experiences. Participants noted that AI helps in reflecting on and assessing outcomes, with one student mentioning:

*Reflective observation, which consists of analyzing and evaluating the experience.*

[Participant 44]

Another shared:

*AI tools allow us to analyse data, simulate scenarios, and create links with known information.*

[Participant 43]

#### Abstract Conceptualization

This was the most cited phase, with 21 responses. AI tools were recognized for aiding in the development of theories from observations. One respondent remarked, underscoring their role in synthesizing and conceptualizing new ideas:

*I use AI tools to understand things, to learn from existing theories, and to develop concepts to integrate the topics*.

[Participant 14]

#### Active Experimentation

Nearly 19 respondents associated AI tools with this phase, where concepts are tested in new situations. AI facilitates this by enabling users to apply and test theoretical concepts practically. One student noted:

*Active experimentation when testing what AI can do and its limits.*

[Participant 50]

#### AI Does Not Fit Well Into Kolb’s Model

A minority of responses (n=3) expressed skepticism about AI’s fit within Kolb’s phases, citing challenges in aligning AI functionalities with the model: “uncertain/did not understand the question.” Two respondents were unsure or confused about how to position AI tools within Kolb’s model.

### Theme 3.4: Skills Required for Effective AI Use in Learning

Several essential skills were identified by the respondents for using AI tools effectively in learning. Technical competency is essential, as 22 respondents highlighted the necessity of comprehending the operation of AI systems, particularly the proper navigation of their features and settings to enhance learning outcomes.

Critical thinking and evaluation skills were also highlighted by 19 respondents, who emphasized the importance of analyzing AI outputs and ensuring they align with established knowledge or academic standards. This is particularly important given the potential for AI to produce inaccurate or incomplete information. One participant explained:

*A variety of skills are needed, critical thinking and good judgement being very important as certain outputs generated by AI can be incorrect or misleading and knowing when to distinguish that is important.*

[Participant 51]

And another participant stated:

*Critical thinking and judgment are essential to determine whether the AI’s response is appropriate in each situation. We must be able to detect biases (what the AI might have forgotten to consider, such as emotions, etc.).*

[Participant 12]

Adaptability and flexibility are key, according to 14 respondents, who noted that users must be open to experimenting with AI and adjusting their strategies as they learn. One respondent stated:

*...Flexibility is also important because you might need to make changes to your prompt to get a desired response from AI.*

[Participant 36]

Effective communication was another important skill, with 12 respondents stressing the need to ask AI clear and specific questions to receive the most useful responses. As one respondent pointed out:

*...knowing how to word questions properly to get the answer you need.*

[Participant 27]

Finally, digital literacy was mentioned by 10 respondents, who recognized not only the importance of staying up to date with AI tools but also the need to use them responsibly and with academic integrity. As one student mentioned:

*Being responsible when processing and using information. Maintaining academic integrity.*

[Participant 2]

### Theme 4: Peer Training and Awareness Strategies

#### Theme 4.1: Methods Used to Learn AI Tools

Health professions students used various methods to learn how to use AI tools. Self-directed exploration was the most common approach, with 23 respondents reporting that they learned by trying out different features and experimenting with the tools independently. One respondent said, which reflects a hands-on approach to mastering AI:

*I just used trial and error.*

[Participant 38]

Social learning also played a role, with 13 respondents indicating that they learned through friends, colleagues, or professors. These students benefited from observing others use AI or receiving direct guidance. This highlights the importance of peer-to-peer learning and mentorship in mastering new technologies. One student noted:

*I heard about the tools from some of my friends and asked them for tips.*

[Participant 33]

Online tutorials and guides were also used by 11 respondents, who turned to YouTube videos, articles, or AI tool help sections to gain a better understanding. As one student shared:

*...I also saw a couple videos on TikTok about using select sentences/words for my desired effect.*

[Participant 34]

Formal training, however, was mentioned by only two respondents, indicating that most AI learning happens informally and independently. One student explained:

*I didn't need to learn anything, ChatGPT was easy to use.*

[Participant 9]

Overall, students adopted diverse strategies for learning use of AI tools, with a clear preference for self-guided exploration and peer learning, supplemented by online resources.

#### Theme 4.2: Best Strategies for Educating and Training Peers in AI Use

To effectively educate and train peers in using AI tools, several strategies were highlighted by the respondents.

Practical, hands-on demonstrations were the most suggested approach, with 26 respondents emphasizing the importance of showing how the tools work in real time. For example, one respondent suggested:

*I think concrete exercises would be more useful. For example, using it as part of a specific group project.*

[Participant 15]

Workshops and guided sessions were also popular recommendations, with 19 respondents advocating for structured opportunities to learn AI in a collaborative environment. These sessions would allow peers to ask questions, practice using the tools, and receive feedback. As mentioned by one student:

*Workshops and small group discussion sessions are the best.*

[Participant 29]

And another elaborated:

*I think workshops could be helpful as it allows for discussion on how to use these AI tools properly and effectively. It also allows for troubleshooting and working together to solve a problem in AI tools.*

[Participant 27]

Providing easy-to-follow tutorials and online resources was another suggested strategy, with 15 respondents supporting the use of videos, articles, or step-by-step guides that could be shared among peers. One respondent explained:

*Self-study modules that students can do at their own pace and learn new ways that can help use AI tools in a smart yet efficient way, without harming our education.*

[Participant 45]

This method would allow for self-paced learning and continuous reference.

Lastly, creating supportive peer networks was recommended by 10 respondents, who emphasized the value of mentorship and peer-to-peer assistance in AI education. This strategy builds a community of learners who can share insights, troubleshoot issues, and offer mutual encouragement. One student stated:

*I think the best strategy is through discussion/exposure by peers/colleagues...This is most appropriate because the discussion aspect primes you to begin thinking about it, so when you approach it individually, you can play around with different aspects based on what you need/want to pursue.*

[Participant 23]

## Discussion

### Principal Findings

Our study collected data from 51 health science students through semistructured interviews and a questionnaire. The findings show the growing adoption of AI tools by students and underscore their importance in academic and clinical settings. Participants reported that general-purpose AI tools, including platforms like ChatGPT, were frequently used. The degree of AI tool use varied depending on the tasks students were required to perform, with some integrating them into their daily activities while others rarely used them. According to participants, AI tools are beneficial for varied learning activities, including rapid information comprehension, facilitating the generation of diverse perspectives, and simulation of varied clinical situations. Students also highlighted the need for collaborative strategies, such as peer learning groups, to promote and ensure effective AI use.

Students identified AI tools as essential to their learning activities. ChatGPT was the most cited tool, while other tools were mentioned only occasionally. This trend reflects the growing use of ChatGPT in health care students’ learning activities, which could be explained by its accessibility, personalized responses, and ability to simulate engaging cognitive interactions. From the perspective of Kolb’s model, this frequent use indicates a significant footprint in the concrete experience phase, while the use of AI to clarify, structure, and reorganize knowledge corresponds to the abstract conceptualization phase. These results are in line with a previous study, which highlighted the ability of ChatGPT to produce logical and contextually relevant explanations, as well as to simulate a logical thinking process, making it a promising didactic tool in the field of education [8,30]

Furthermore, students expressed a preference for using AI tools over traditional search engines like Google Search, due to the more targeted and precise nature of the results. This suggests that students are looking for tools that support conceptual organization and meaning-making, which corresponds to the abstract conceptualization phase of Kolb’s experiential learning cycle. Previous studies confirmed this preference among students for ChatGPT over Google Search, as this tool appears to provide more accurate and comprehensive medical information for general knowledge requests and highlights its usefulness in the educational environment [8,31].

Students reported variability in their use of AI tools, ranging from occasional use to regular integration into their learning routines. This observed inconsistency appears to reflect significant differences in individual usage styles, the familiarity developed with AI technologies, and the nature of the tasks they were faced with. In the context of experiential learning, this variability may also indicate that students do not all engage in the learning cycle in the same way, with some focusing primarily on concrete experience and reflective observation, while others broaden their use to abstract conceptualization and active experimentation by reinvesting AI results in new academic or clinical tasks. These results are in line with recent studies confirming that the degree of use of AI tools depends on their usefulness as well as specific academic contexts and tasks [32,33]. Understanding this nuance is important because it suggests the importance of promoting their use in an educational context by adopting a more individual rather than a universal approach [9,33].

Our results also confirmed that a lot of health care students use ChatGPT to learn and develop new skills, such as improving their writing, analyzing data, and simplifying complex ideas. These practices are part of Kolb’s experiential learning theory and are explained by reflective observation of AI-generated results and abstract conceptualization. These findings are consistent with previous studies, which concluded that ChatGPT improves student writing, leading to more effective and higher-quality academic production [10,34]. Moreover, other more specialized tools used by these students, such as Consensus for synthesizing scientific articles, demonstrate a strategic and targeted use of AI tools to perform specific tasks, aligning with previous studies [35,36]. On the other hand, some students reported not using these technologies, often due to lack of interest or confidence, which is consistent with the writings of Johnston et al [37], who show that students who have greater confidence in their ability to write academic texts use ChatGPT or other generative AI tools less in their learning activities. These results show that efforts are still needed to promote equitable and critical integration of these tools into educational practices, although AI remains an important tool for skill development [37].

In terms of effective strategies for training peers to use AI tools, students identified hands-on demonstrations, guided workshops, and self-directed resources. These findings are part of Kolb’s experiential learning theory and are explained by the importance of concrete experience (hands-on demonstrations), reflective observation (guided discussions and feedback), and active experimentation (autonomous reuse of tools in personal learning contexts). These approaches are consistent with the study of Jiang et al [38], which highlights the importance of active and collaborative learning to foster engagement and understanding in education, as well as with that of Abid et al [39], which demonstrates the effectiveness of flexible and online approaches in technology training. Participants also valued peer support, highlighting the central role of social collaboration and peer support in technology interactions, as suggested by previous studies [14,40]. These findings confirm that effective AI adoption requires a combination of hands-on activities, autonomy, and collaborative support.

### Strengths

One of the strengths of this study is its methodological framework, which facilitated the collection of rich and nuanced opinions from health science students on the use of AI. Next, the study includes individuals with varied experiences using AI tools, both in academic and clinical environments. This highlights not only the different ways in which AI is used, but also the motivations and critical thinking strategies implemented by students. Finally, the highlighted didactic implications are directly applicable. They emphasize the need to prepare students for a critical approach to AI, to encourage tailored learning environments, and to promote the incorporation of AI into collaborative tasks.

### Limitations

One of the limitations of this study is the small sample size and the use of self-reported data, and the fact that the data were collected within a single establishment, which limits the generalization of the results to other institutions. This may have introduced biases related to memorization accuracy or social desirability. Although the research team assessed the questionnaire for relevance and clarity, psychometric procedures were not sufficiently applied to assess it. Therefore, interpretation biases may arise, particularly regarding the understanding of porous concepts such as “learning” or “learning support.” Furthermore, health professions students were the focus of this study; the application of AI in training may vary by discipline. In addition, this transition in data collection, from semistructured interviews to online surveys, may have reduced the depth and richness of some responses, which could affect the identification of more subtle themes.

Therefore, future research could address these limitations by conducting larger-scale studies with more diverse samples and using objective measures of AI usage, such as usage logs or performance data. Additionally, future research could explore how AI is used in other fields of study and whether the patterns observed in this study are generalizable to different educational contexts. Moreover, as AI tools continue to evolve, it will be important to track how students’ perceptions and utilization habits change over time.

### Conclusions

In conclusion, this study aimed to identify the AI tools most used by health students at the University of Ottawa, to determine their usage habits as well as the tools helping them develop skills, and to examine the strategies they consider effective for training their peers in the use of these tools. Our findings highlight the growing role of AI in health care education and provide valuable insights into how these tools have been adopted and used by students. While AI is considered essential to their learning activities, it becomes crucial to further integrate it into educational programs and help students develop their digital skills while developing their critical thinking skills. As these technologies gain popularity, they have the potential to enhance the learning experience of health care students, but careful attention must be paid to how these tools are promoted and used in the educational environment.

## Supplementary material

10.2196/82432 Multimedia Appendix 1 Question guide and distribution of artificial intelligence tools used by health professions students at the University of Ottawa.

10.2196/82432 Checklist 1 SRQR checklist.

## ABBREVIATIONS

AI: artificial intelligence
REB: Research Ethics Board
SRQR: Standards for Reporting Qualitative Research
