# Supplementary material for: Artificial Intelligence in Health Professions Education: Qualitative Study of Student Experiences
Source: J Med Internet Res. 2026 Apr 2;28:e82432. doi: 10.2196/82432 (PMC13045872; doi:10.2196/82432)
Supplement: Multimedia Appendix 1 [file jmir-v28-e82432-s001.pdf]

Question guide and distribution of AI tools used by health professions students at the University of Ottawa

A - Question guide (interviews and questionnaire)

1- I agree to participate in this research project led by Dr. Fotsing and his team. I understand that by clicking "I accept" and completing and submitting the online questionnaire, I agree to participate in this research project.

2- What is your age range?

3- What is your gender identity?

4- What is your field of study?

5- Could you please tell us about the AI tools you use most often? Many healthcare students use platforms such as Med Chatbot, ChatGPT, and Google AI Healthcare in their learning. In your case, which ones do you use?

6- Do you consider AI to be an essential component of your learning, and if so, why?

7- What prompted you to start using AI tools?

8- Utilisation Habits: What are your utilisation habits with regards to these tools? How often do you use them? Is your usage consistent or sporadic?

9- What exactly do you use AI tools for? How do you use these tools? For example, to write, revise, learn, synthesize information, test your knowledge, other?

10- How do you apply critical reasoning to judge the quality of the information you receive when using AI tools? (i.e. do you apply critical reasoning to judge the quality of the information you receive when using AI tools?)

11- According to you, which AI tools do you think have enabled you to acquire new knowledge and develop skills?

12- Did you try out several AI tools before settling on the one you're using now?

13- If so, why did you choose the tool you're using now (e.g. advantages, disadvantages, loyalty, etc.)?

14- In connection with Vignette 1 (see below), in your opinion, where do AI tools fit in the definition of learning, and why?

Vignette 1: Learning is a process that enables the individual to perceive objects, interact with them, and integrate them with their social, cognitive, and affective dimensions to transform, create, or develop their cognitive structure. The objects of learning are information, knowledge, skills, attitudes, and values.

15- In connection with Vignette 2 (see below), which explains experiential learning according to Kolb, where do you situate the use of AI tools in your learning and why?

Vignette 2: Kolb (1984) divides the experiential learning process into four phases: - Concrete experience, which consists of experiencing a new situation or revisiting a known one. - Reflective observation, which consists of analyzing and evaluating the experience. - Abstract conceptualization, which consists of developing concepts, principles, or theories based on observation. - Active experimentation, which consists of testing and applying concepts, principles, or theories in new situations.

16- To use various AI tools, did you undergo any prior training? For example, have you taken courses, read articles, watched online videos, learned via social media, etc.?

17- If so, can you describe which training you took?
18- In your opinion, did this training prepare you well to use AI in your learning?
19- If you didn't take any training, how did you go about learning how to effectively use AI?
20- What do you think are the best strategies for educating and training your peers in the proper use of AI in their learning? There are several methods to train students and help them acquire new knowledge and develop new skills, such as lectures, self-study modules, workshops, small group discussion sessions, etc. Which do you think would be more appropriate and why?
21- In your opinion, what skills are required to use AI effectively in learning? (e.g. critical thinking, analytical judgment, flexibility, intellectual curiosity, bias detection and management, delegation, etc.)
22- If you wish to participate in the draw, please enter your e-mail address.

B- Distribution of AI tools used by health professions students at the University of Ottawa.

AI tools	Number of students who used these tools
ChatGPT	49
Microsoft Copilot	2
Perplexity AI	2
Claude	2
Google Bard AI	1
Mind Grasp	1
Explain Like I'm 12	1
Summarized AI	1
Word tune AI	1
Grammarly AI	1
Midjourney	1
Soundraw	1

AI tools	Number of students who used these tools
Eleven Labs	1
Snapchat AI	1
Aura AI	1
SCITE	1
Consensus	1
DeepL	1
Research Rabbit	1
OpenAI	1
Google AI	1
Bing AI	1
Bing Chat	1
AskYourPDF	1
Leonardo	1
